# Hereditary Tumours

**DOI:** 10.1155/2013/490357

**Published:** 2013-05-14

**Authors:** Francesco Baudi

**Affiliations:** ^1^Dipartimento di Medicina Sperimentale e Clinica, Universitá “Magna Graecia” di Catanzaro, 88100 Catanzaro, Italy; ^2^Centro Oncologico Fondazione T. Campanella, 88100 Catanzaro, Italy

Hereditary tumours account for 3–10% of all cancers. Greater susceptibility of the subjects to these types of malignancies is thought to be the result of genetic mutations in highly penetrant genes transmitted as autosomal dominant traits. Mutations in such susceptibility genes result in familial predisposition to developing a characteristic spectrum of diseases (collectively known as Hereditary Cancer Syndromes). While these mutations do not cause the disease *per se*, their carriers exhibit a higher risk (usually ≥50%) of developing the disease during their lifetime.

Most of the studies carried out in this field have focused on the most common hereditary tumours, breast cancer and colorectal cancer, for which the major susceptibility genes, BRCA1 and BRCA2 and MLH1 and MSH2, respectively, were identified about 20 years ago. The discovery of these genes has made it possible to translate, in a relatively short amount of time, basic research findings into clinical practices (bench to bedside) by contributing appropriate diagnostic strategies. These include genetic tests that are currently used to screen families for the identification of mutation carriers and to improve both prevention and management of hereditary cancers. At the same time, the study of cancer-specific signaling pathways and pathogenetic mechanisms has allowed the development of targeted therapies.

In this special issue, P. Apostolou and F. Fostira provide an overview on the current knowledge of the genes causing predisposition to breast cancer. Besides BRCA1 and BRCA2, the most frequently mutated genes, the authors also review the contribution of additional genes to hereditary breast cancer syndromes that have recently emerged through whole-genome analyses. Mutations in these newly discovered genes account for the heterogeneity found among different syndromes and result in differential diagnoses.

About one hundred syndromes characterized by Mendelian inheritance can be ascribed to the presence of very rare alleles [1], yet these rare alleles are found in only 20–40% of cancers where inheritance is suspected. Given this paradox, other mechanisms must be at play, such as the presence of specific genomic structural variants that might escape common methods of detection based on Sanger sequencing. In their research article, F. Duraturo et al. show the application of multiplex ligation-dependent probe amplification (MLPA) for determining the contribution of large genomic rearrangements of MLH1 and MSH2 genes in a population of Lynch syndrome patients where analysis for point mutations was previously negative. The MLPA allows to retrieve an even small percentage of “missing heritability” due to structural variants other than classic substitutions or small indels. We can expect that, when the actual contribution of these structural variants to various diseases is elucidated, we will be able to perform advanced genetic testing, including a more comprehensive evaluation of additional structural variants, such as large-scale copy number variation (CNV) or epimutations.

The deterministic model, which attributes an inherited susceptibility to mutations in high-penetrance cancer-specific genes, has been joined more recently by an alternative “polygenic” model that is based on the cumulative role of multiple low-penetrance alleles [2]. Many of these loci have been identified by genome-wide association (GWA) studies and a list of those involved in susceptibility to breast cancer is reviewed by P. Apostolou and F. Fostira. An estimation of the penetrance of these rare alleles and their corresponding risk is complicated; in turn, this makes genetic counselling very difficult especially in cases where prevention strategies are based on prophylactic surgery. The advent of novel sequencing technologies, from whole-genome to exome-sequencing, will likely accelerate progress in this field.

Since cancer is characterized by a peculiar gene expression profile, another mechanism that could explain “missed heritability” is posttranscriptional regulation. The discovery of microRNAs (miRNAs), small noncoding single-stranded RNA molecules that act as master switches in the regulation of gene expression, has expanded the research on cancer susceptibility to these RNAs. miRNA-mediated silencing may be shaping the characteristic gene expression signatures of different cancers and defining tumour subtypes. R. Iuliano et al. summarize the main effect on tumour susceptibility of single-nucleotide polymorphisms (SNPs) included in regions related to miRNA-dependent pathways. SNPs are categorized into three main divisions: SNPs that are mapped within miRNA genomic regions and may participate in carcinogenesis by altering the expression of tumour-related microRNAs, SNP variants located in 3′-UTRs of miRNA-targeted genes that can influence tumour susceptibility by destroying or creating miRNA binding sites, and SNPs in genes encoding microRNA-processing complexes that influence the expression of proteins involved in the miRNA biogenesis pathway.

The expansion of the catalog of variants and the redefinition of risk factors that will ensue should be integrated with a deeper knowledge of the consequences on the pathways that these genes control in order to design new, more efficient strategies for prevention and therapy. A useful contribution to the understanding of the mechanisms of tumour susceptibility may also come from the study of rare cancers, as well demonstrated by the review of Y. Nibu et al. Here, the authors describe the molecular mechanisms underlying notochord formation and the onset of chordoma, a rare bone cancer for which sporadic as well as hereditary forms are known. The review is focused on the *Brachyury* gene, which is the main regulator of the notochord formation, a causative agent of chordoma and a potential therapeutic target. The authors describe a number of potential mechanisms of action of this gene during tumourigenesis, ranging from modulation of a series of genes downstream of this transcription factor, the dosage-dependent effects on them, the interaction of *Brachyury* with other proteins that may affect its function, up to molecules and signaling pathways that regulate its expression. The implication of a developmental gene in the pathogenesis of the disease supports the cancer stem cell hypothesis that may open the search for potential therapies targeting *Brachyury*.

In cancers showing a familial component without known inheritable causes and satisfying disease management, the implementation of appropriate preventive measures at an early age for people considered to be at higher risk is mandatory. Particular attention should be given to subjects with family history of cancer without tractable mutations in major susceptibility genes. These issues are well explained in the manuscript of G. Corso which addresses the issue of familial intestinal gastric cancer (FIGC). Since the genetic basis of susceptibility to this cancer is largely unknown, careful protocols for monitoring and management of asymptomatic patients have to be developed. The proposed management flowchart follows the presentation of a very original clinical case centered on the family history of gastric cancer of the Roncalli family, the family of Pope John XXIII, the Pope of the Catholic Church who died in 1963 of peritonitis resulting from perforation of a gastric cancer lesion.
